# Supplementary material for: Mapping the evolution of fertility support policies in China: A content and instrumental analysis
Source: PLoS One. 2025 Oct 9;20(10):e0332137. doi: 10.1371/journal.pone.0332137 (PMC12510515; doi:10.1371/journal.pone.0332137)
Supplement: S1 Appendix — (ZIP) [file pone.0332137.s001.zip › S1 Appendix. 226 original policy documents/31-国家卫生健康委办公厅、中央编办综合局、民政部办公厅、市场监管总局办公厅关于印发托育机构登记和备案办法(试行)的通知(FBM-CLI-4-338643).doc]

国家卫生健康委办公厅、中央编办综合局、民政部办公厅、市场监管总局办公厅关于印发托育机构登记和备案办法(试行)的通知

**发布部门：** [国家卫生健康委员会](https://www.pkulaw.com/law/chl?Aggs.IssueDepartment=60321) [中央机构编制委员会办公室](https://www.pkulaw.com/law/chl?Aggs.IssueDepartment=a31) [民政部](https://www.pkulaw.com/law/chl?Aggs.IssueDepartment=60208) [国家市场监督管理总局](https://www.pkulaw.com/law/chl?Aggs.IssueDepartment=60426) [机构沿革](javascript:void(0))

**发文字号：**国卫办人口发〔2019〕25号

**发布日期：**2019.12.19

**实施日期：**2019.12.19

**时效性：** [现行有效](https://www.pkulaw.com/law/chl?Aggs.TimelinessDic=01)

**效力级别：** [部门规范性文件](https://www.pkulaw.com/law/chl?Aggs.EffectivenessDic=XE0302)

**法规类别：** [机关工作综合规定](https://www.pkulaw.com/law/chl?Aggs.Category=00301)

国家卫生健康委办公厅、中央编办综合局、民政部办公厅、市场监管总局办公厅关于印发托育机构登记和备案办法（试行）的通知
（国卫办人口发〔2019〕25号）

各省、自治区、直辖市及新疆生产建设兵团卫生健康委、编办、民政厅（局）、市场监管局（厅、委）：
　　根据《[国务院办公厅关于促进3岁以下婴幼儿照护服务发展的指导意见](https://www.pkulaw.com/chl/434b24b8b5769383bdfb.html)》（国办发〔2019〕15号）要求，为规范托育机构的登记和备案管理，国家卫生健康委办公厅、中央编办综合局、民政部办公厅、市场监管总局办公厅制定了《托育机构登记和备案办法（试行）》（可从国家卫生健康委网站下载）。现印发给你们，请遵照执行。

国家卫生健康委办公厅
中央编办综合局
民政部办公厅
市场监管总局办公厅
2019年12月19日

　　托育机构登记和备案办法（试行）

　　**第一条**　为贯彻落实《[国务院办公厅关于促进3岁以下婴幼儿照护服务发展的指导意见](https://www.pkulaw.com/chl/434b24b8b5769383bdfb.html)》（国办发〔2019〕15号）精神，规范托育机构的登记和备案管理，依据《[国家卫生健康委关于印发托育机构设置标准（试行）和托育机构管理规范（试行）的通知](https://www.pkulaw.com/chl/bc9a2a9250f37a24bdfb.html)》（国卫人口发〔2019〕58号）及相关规定，制定本办法。

　　**第二条**　本办法适用于为3岁以下婴幼儿提供全日托、半日托、计时托、临时托等服务的托育机构。

　　**第三条**　举办托育机构的，应当按照本办法规定办理登记和备案。
　　法律、行政法规另有规定的，依照有关规定执行。

　　**第四条**　举办事业单位性质的托育机构的，向县级以上机构编制部门申请审批和登记。
　　举办社会服务机构性质的托育机构的，向县级以上民政部门申请注册登记。
　　举办营利性托育机构的，向县级以上市场监督管理部门申请注册登记。

　　**第五条**　托育机构申请登记时，应当在业务范围（或经营范围）中明确托育服务内容。
　　托育机构申请登记的名称中可包含“托育”字样。

　　**第六条**　登记机关应当及时将托育机构登记信息通过共享、交换等方式推送至同级卫生健康部门。

　　**第七条**　县级卫生健康部门负责辖区内已登记托育机构的备案。

　　**第八条**　托育机构应当及时向机构所在地的县级卫生健康部门备案，登录托育机构备案信息系统，在线填写托育机构备案书、备案承诺书，并提交以下材料扫描件：
　　（一）营业执照或其他法人登记证书；
　　（二）托育机构场地证明；
　　（三）托育机构工作人员专业资格证明及健康合格证明；
　　（四）评价为“合格”的《托幼机构卫生评价报告》；
　　（五）消防安全检查合格证明；
　　（六）法律法规规定的其他相关材料。
　　提供餐饮服务的，应当提交《食品经营许可证》。

　　**第九条**　卫生健康部门在收到托育机构备案材料后，应当在5个工作日内提供备案回执和托育机构基本条件告知书。
　　卫生健康部门发现托育机构备案内容不符合设置标准和管理规范的，应当自接收备案材料之日起15个工作日内通知备案机构，说明理由并向社会公开。

　　**第十条**　托育机构变更登记、注销登记后，应当及时登录托育机构备案信息系统向卫生健康部门变更备案信息或报送注销信息。

　　**第十一条**　卫生健康、编制、民政、市场监管等部门应当将托育服务有关政策规定、托育机构登记和备案要求、托育机构有关信息在官方网站公开，接受社会查询和监督。

　　**第十二条**　省级卫生健康、编制、民政、市场监管部门可结合当地实际情况制定实施细则。

　　**第十三条**　本办法自印发之日起施行。

© 北大法宝：（[www.pkulaw.com](https://www.pkulaw.com/)) 专业提供法律信息、法学知识和法律软件领域各类解决方案。北大法宝为您提供丰富的参考资料，正式引用法规条文时请与标准文本核对。
欢迎查看所有[产品和服务](https://www.pkulaw.com/helps/2.html)。法宝快讯：[如何快速找到您需要的检索结果？](https://www.pkulaw.com/helps/70.html)[法宝](https://www.pkulaw.com/helps/69.html)V6有何新特色？

扫描二维码阅读原文

**原文链接：**<https://www.pkulaw.com/chl/85a4eed3fc0e799cbdfb.html>
